# Synthesis and Anti-Bacterial Activities of a Bis-Chalcone Derived from Thiophene and Its Bis-Cyclized Products

**DOI:** 10.3390/molecules16010523

**Published:** 2011-01-12

**Authors:** Abdullah M. Asiri, Salman A. Khan

**Affiliations:** 1 Chemistry Department, Faculty of Science, King Abdul Aziz University, P.O. Box 80203, Jeddah 21589, Saudi Arabia; 2 The Center of Excellence for Advanced Materials, King Abdul Aziz University, P.O. Box 80203, Jeddah 21589, Saudi Arabia

**Keywords:** chalcone, pyrazoline, pyrimidine, anti-bacterial activity, chloramphenicol

## Abstract

A chalcone was prepared by the reaction of terephthalaldehyde with 3-acetyl-2,5-dimethylthiophene. Treatment of this chalcone with thiosemicarbazide/phenyl hydrazine/guanidine hydrochloride/thiourea afforded the corresponding pyrazoline, pyrazole, and pyrimidine in good yields. All the new compounds have been characterized by IR, ^1^H-NMR, ^13^C-NMR, GC-MS and elemental analyses. The anti-bacterial activity of these compounds were first tested *in vitro* by the disk diffusion assay against two Gram-positive and two Gram-negative bacteria, and then the minimum inhibitory concentration (MIC) was determined with the reference of standard drug chloramphenicol. The results showed that the pyrazoline derivative is better at inhibiting growth of both types of bacteria (Gram-positive and Gram-negative) compared to chloramphenicol.

## 1. Introduction

α,β-Unsaturated ketones are biogenetic precursors of flavonoids in higher plants. Also known chemically as chalcones, they consist of open-chain flavonoids in which the two aromatic rings are joined by a three carbon chain [1]. They display a wide range of pharmacological properties, including cytotoxity towards cancer cell lines [2,3], antimitotic [4], antimutagenic [5] and antitumor-promoting activities; antibacterial [6], antiviral [7], anti-inflammatory [8], antiulcerative [9] and hepatoprotective activities [10]. They are also useful in materials science fields such as non-linear optics (NLO) [11], optical limiting [12], electrochemical sensing [13], Langmuir films and photoinitiated polymerization [14]. Various chalcone derivatives are notable materials for their second harmonic generation (SHG) [15]. They are well known intermediates for synthesizing various heterocyclic compounds. Cyclization of chalcones, leading to thiazines, pyrimidines, pyrazline has been a developing field within the realm of heterocyclic chemistry for the past several years because of their ready accessibility and the broad spectrum of biological activity of the products as antibacterial, antifungal, antiprotozoal, anti-inflammatory substances. A survey of literature in the recent past reveals that some pyrazoline derivatives possess antibacterial [16], anti-inflammatory [17], and antifungal effects [18]. Thiazine derivatives play a vital role in many biological processes and in the synthesis of drugs [19]. Pyrimidine derivatives occur in natural products like nucleic acids and vitamin B1 and they have remarkable pharmaceutical importance because of their biological activity as anti HIV, antitubercular, and antidiabetic compounds [20,21]. These observations led us to synthesize a new bis-chalcone and its corresponding bis-pyrazoline, bis-pyrazole and bis-pyrimidines and an examination of their antibacterial properties.

## 2. Results and Discussion

### 2.1. Chemistry

**Scheme 1:**
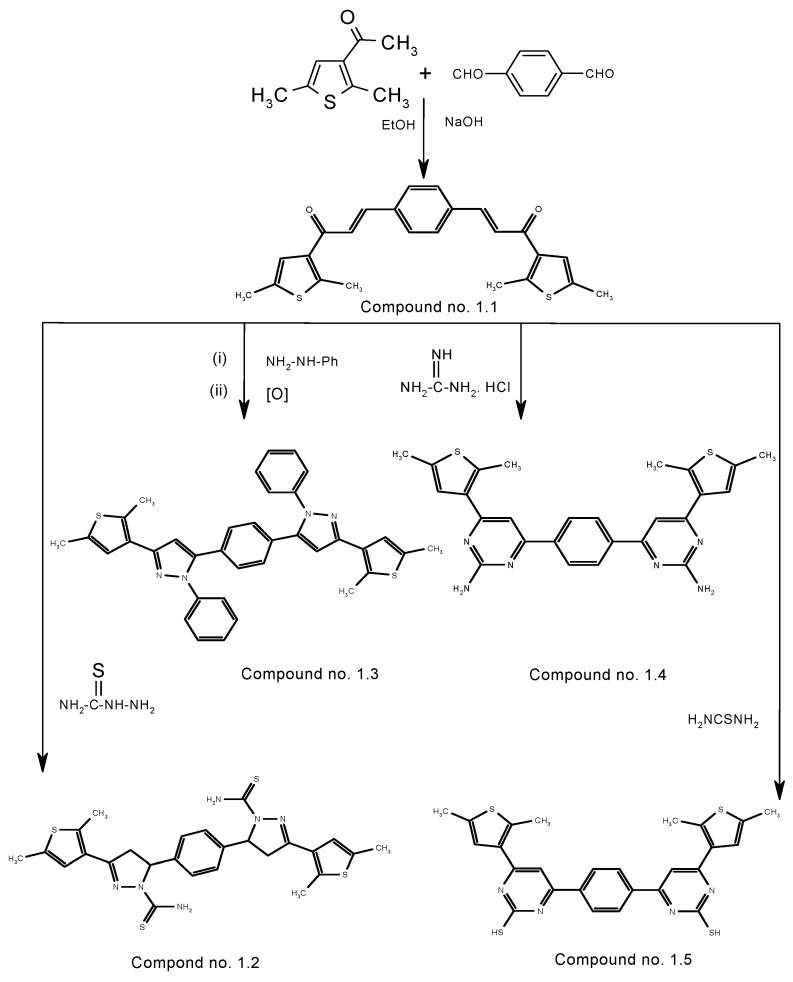
The synthesis of the chalcone and its cyclized products.

In the present work, the cyclization of a bis-chalcone into the corresponding bis-pyrazoline, bis-pyrazole and bis-pyrimidine derivatives was accomplished by the reaction of the chalcone with thiosemicarbazide /phenyl hydrazine/guanidine hydrochloride/thiourea [22,23,24]. The synthetic route to the compounds is outlined in Scheme 1. The chemical structures of the synthesized compounds were established by spectroscopic data (FT-IR, ^1^H-NMR, ^13^C-NMR, GC-MS) and elemental analyses.

The IR spectrum of compound **1.1** showed the characteristic band at 1,648 cm^-1^ which indicates the presence of a –C=O group. The IR spectrum of compound **1.2** showed the characteristic bands at 1,578 cm^-1^ and 1,361 cm^-1^ which indicate the presence of –C=N and C=S groups. The IR spectrum of compound **1.3** also showed characteristic bands at 1,657 cm^-1^ and 1,594 cm^-1^ which indicate the presence of –C=C and C=N groups. The IR spectrum of compound **1.4** showed characteristic bands at 3,323 cm^-1^ and 1,566 cm^-1^ indicative of the presence of NH_2_ and C=N groups. The IR spectrum of compound **1.5** showed characteristic bands at 1,134 cm^-1^ and 678 cm^-1^ which indicate the presence of C-N and C-S groups.

The structures of the chalcone and its cyclized products were further confirmed by the corresponding ^1^H-NMR spectra. The ^1^H-NMR spectrum of compound **1.1** showed two doublets at 7.20 ppm (*J* = 15.3 Hz) and 8.04 ppm (*J* = 15.3 Hz), indicating that the ethylene moiety in the enone linkage is in a *trans*-conformation in the chalcone. The ^1^H-NMR spectrum of compound **1.2** showed a –CH_2_ multiplet at 3.02-5.85 ppm confirming the cyclisation of the chalcone to give pyrazoline **1.2**. The ^1^H-NMR spectrum of compound **1.3** showed a singlet at 6.50 ppm due to CH=C protons and no peak in the 3.05 5.85 ppm range, which indicates the oxidation of the pyrazoline in the reaction and its conversion to a pyrazole.

The ^1^H-NMR spectrum of compound **1.4** showed a sharp singlet at δ 8.12 due to the NH_2_ protons, and it also showed a sharp singlet at δ 7.13 due to HC=C, which confirmed the cyclization of the chalcone into a pyrimidine ring. The ^1^H-NMR spectrum of compound **1.5** shows a sharp singlet at δ 3.37 due to the S-H protons, and also a sharp singlet at δ 7.23 due to HC=C, confirming the cyclisation of the chalcone to give a pyrimidine ring. Finally, the ^13^C-NMR spectra of the chalcone and the cyclized producta were recorded in DMSO-d_6_ and the spectral signals were in good agreement with the proposed structures. Details of the ^13^C-NMR spectra of all compounds are given in the Experimental Section. Characteristic molecular ion peaks were observed in the mass spectra of the chalcone and the cyclized products. For example, the spectrum of compound **1.5** shows a molecular ion peak (M^+.^) at *m/z* 520.

### 2.2. Pharmacology

The *in vitro* antibacterial activity tests were performed using the disk diffusion method and the Minimum Inhibitory Concentration (MIC) method. Chloramphenicol was used as positive control for bacteria. The compounds **1.1-1.5** were tested for their antibacterial activities by disc-diffusion method [25] using nutrient broth medium [containing (g/L): beef extract 3 g; peptone 5 g; pH 7.0]. The Gram- positive bacteria and Gram-negative bacteria utilized in this study were *S. aureus*, *S. pyogenes*, *S. typhimurium* and *E. coli*. In the disc-diffusion method, sterile paper discs (0.5 mm) impregnated with compound dissolved in dimethylsulfoxide (DMSO) at a concentration of 100 μg/mL were used. Then, the paper discs impregnated with the solution of the compound tested were placed on the surface of the media inoculated with the microorganism. The plates were incubated at 35 °C for 24 h. The growth inhibition zones after incubation are shown in **Table 1**. The chalcone and its cyclized products were further checked by the MIC method. The results are presented in **Table 2**. Among the five compounds the pyrazoline derivative showed better anti-bacterial activity than the control drug chloramphenicol. The distinct differences in the antibacterial properties of these compounds further justify the purpose of this study. The importance of this work lies in the possibility that the new compound might be more efficacious drugs against bacteria and a more thorough investigation regarding the structure–activity relationships, toxicity and in their biological effects could be helpful in designing more potent antibacterial agents for therapeutic use.

## 3. Experimental 

### 3.1. General

All the chemicals and solvents used for this work were obtained from Merck (Germany) and the Aldrich Chemical Company (U.S.A.). Melting points of the synthesized compounds were determined in open-glass capillaries on a Stuart-SMP10 melting point apparatus and are uncorrected. IR absorption spectra were recorded in the 4,000–400 cm^-1^ range on a Shimadzu FTIR-8400s using KBr pellets, ^1^H-NMR and ^13^C-NMR spectra were recorded on a Bruker-AVANCE-III 600 MHz spectrophotometer. The ^1^H-NMR and ^13^C-NMR chemical shifts are reported as parts per million (ppm) downfield from TMS (Me_4_Si) used as an internal standard. The splitting patterns are designated as follows; s, singlet; d, doublet; m, multiplet. Mass spectra were recorded on an GC-MS spectrometer. IR, ^1^H-NMR, ^13^C-NMR and MS data were consistent with the assigned structures. Elemental analyses (C, H, N) were done on a CHN Rapid analyzer. All the new compounds gave C, H and N analysis within ±0.03% of the theoretical values. Purity of the compounds was checked by thin layer chromatography (TLC) on Merck silica gel 60 F254 precoated sheets eluted with a chloroform/methanol mixture and spots were developed using iodine vapours/ultraviolet light as visualizing agent.

*(2E,2'E)-3,3-(1,4-Phenylene)bis[1-(2,5-dimethyl-3-thienyl)prop-2-en-1-one]* (**1.1**). A solution of 3-acetyl-2,5-dimethylthiophene (0.029 mol) and terephthalaldehyde (2 g, 0.014 mol) in an ethanolic solution of NaOH (6 g in 10 mL of ethanol) was stirred for 20 h at room temperature. The solution was poured into ice cold water of pH~2 (pH adjusted by HCl). The solid was separated and dissolved in CH_2_Cl_2_, washed with saturated solution of NaHCO_3_ and evaporated to dryness. The residue was purified by column chromatography using CH_2_Cl_2_ as eluent. Yield: 78%; m.p. 194-195 °C. Anal. calc. for C_24_H_22_O_2_S_2_: C, 70.91, H, 5.41, S, 15.57, Found: C, 70. 86, H, 5.35, S, 15.52; GC-MS *m/z* (rel. int.%): 407 (40) [M+1]^+^, 255 (70), 153 (45); IR (KBr) *v*max cm^-1^: 3,050 (Ar-H), 2,914 (C-H), 1,648 (C=O), 1,585 (C=C); ^1^H-NMR (DMSO-*d*6) (δ/ppm): 7.71 (d, 2H, *J* = 15.6 Hz, C=CH), 7.30 (d, 2H, *J* = 15.6 Hz, CO=CH), 7.64 (s, 4H, Ar-H), 7.09 (s, 2H, thiophene-H), 1.61 (s, CH3);^13^C-NMR (DMSO-*d*6) (δ/ppm): 186.09, 147.77, 142.36, 136.77, 136.47, 135.46, 130.21, 125.76, 15.98, 15.07.

### 3.2. Synthesis of pyrazoline ***1.2*** from thiosemicarbazide

A mixture of bis-chalcone **1.1** (0.004 mol), thiosemicarbazide (0.009 mol) and NaOH (0.002 mol) in dry ethanol (30 mL) was refluxed at 80 ºC for 12 h. The progress of reaction was monitored by TLC. After the completion of reaction, the reaction mixture was poured into acidic ice water (~ pH 2, adjusted by HCl). The solid was filtered off and the residue was purified by column chromatography (20:80, diethyl ether-petroleum ether). The obtained solid was crystallized from ethanol. Yield: 72.6%; m.p. 236 °C; Anal. calc. for C_26_H_28_N_6_S_4_: C, 56.49, H, 5.11, N, 15.20, Found: 56.45, H, 5.09, N. 15.18; GC-MS *m/z* (rel. int.%): 554 (40) [M+1]^+^; IR (KBr) *v*_max_ cm^-1^: 3,436 (NH), 3,253 (Ar-H), 2,960 (C-H), 1,578 (HC=N), 1,361 (C=S), 1,092 (C-N); ^1^H-NMR (DMSO-*d*_6_) (δ/ppm): 7.98 (s, 4H, NH_2_), 7.51 (s, 4H, Ar-H), 7.07 (s, 2H, thiophene-H), 5.85 (dd, 2H, H_x_, *J_XA_* = 3.0 Hz, *J_XB_* = 3.6 Hz), 3.90 (dd, 2H, H*_A_*, *J_AB_* = 3.0 Hz, *J_AX_* = 3.6 Hz), 3.02 (dd, 2H, H*_B_*, *J_BA_* = 3.0 Hz, *J_BX_* = 3.6 Hz), 2.55 (s, CH3), 2.46 (s, CH_3_);^13^C- NMR (DMSO-*d*6) (δ/ppm): 175.46 (C=S), 152.39 (C=N), 141.32, 135.17, 127.70, 126.38, 125.39 (Ar-C), 61.60 (CH), 44.39 (CH_2_), 15.42 15.41 (CH_3_).

### 3.3. Synthesis of pyrazole ***1.3*** from phenyl hydrazine

*Bis-Chalcone* **1.1** (0.004 mol) was refluxed with phenyl hydrazine (0.009 mol) in dry EtOH (20 mL) and a catalytic amount of glacial acetic acid at 80 ºC for 8 h. The progress of the reaction was monitored by TLC. After completion of the reaction, the solvent was removed under reduced pressure and the residue was purified by column chromatography (20:80, diethyl ether-petroleum ether). The obtained solid was crystallized from ethanol. Yield: 88.5%; m.p. 134 °C; Anal. calc. for C_36_H_30_N_4_S_2_: C, 74.19, H, 5.19, N, 9.61, Found: C, 74.15, H, 5.16, N, 10.97; GC-MS *m/z* (rel. int.%): 584 (72) [M+1]^+^; IR (KBr) *v*_max_ cm^-1^: 3,258 (Ar-H), 2,956 (C-H), 1,657 (C=C), 1,594 (HC=N), 1,003 (C-N); ^1^H-NMR (DMSO-*d*_6_) (δ/ppm): 7. 29 (m, 10H, Ar-H), 7.22 (s, 2H, thiophene-H), 6.50 (s, 2H, C=CH), 7.28 (s, 4H, Ar-H), 2.50 (s, CH3), 2.24 (s, CH_3_);^13^C-NMR (DMSO-*d*6) (δ/ppm): 147, 146, 144, 143, 138, 136, 131, 129, 128, 126, 125, 121, 113, 112, 112, 15.38, 13.90.

### 3.4. Synthesis of pyrimidine ***1.4*** from guanidine hydrochloride

A mixture of chalcone **1.1** (0.004 mol), guanidine hydrochloride (0.009 mol) and sodium methoxide (0.004 mol) in DMF (15 mL) was refluxed at 80 °C for 30 h. The progress of reaction was monitored by TLC. After the completion of reaction, the reaction mixture was poured into ice water to give a precipitated solid that was filtered off. The residue was purified by column chromatography (40:50, diethyl ether-petroleum ether) and the obtained solid was crystallized from ethanol. Yield: 79.52%; m.p. 123 °C; Anal. calc. for C_26_H_24_N_6_S_2_: C, 64.44, H, 4.99, N, 17.34, Found: C, 64.41, H, 4.96, N, 17.29; GC-MS *m/z* (rel. int.%): 486 (56) [M+1]^+^; IR (KBr) *v*_max_ cm^-1^: 3,323 (NH_2_), 2,919 (C-H), 1,566 (C=N); ^1^H-NMR (DMSO-*d*_6_) (δ/ppm): 8.12 (s, 4H, NH_2_), 7.26 (s, 4H, Ar-H), 7.13 (s, 2H, Ar-Pym), 7.04 (s, 2H, thiophene-H), 2.45 (s, CH3), 2.17 (s, CH_3_);^13^C-NMR (DMSO-*d*6) (δ/ppm): 139.49 (C=N), 139.49, 136.05, 135.20, 128.86, 127.18, 126.05, 125.32. 141.32, 135.17, 127.70, 126.38, 125.39 (Ar-C), 15.25 15.01 (CH_3_).

### 3.5. Synthesis of pyrimidine ***1.5*** from thiourea

A mixture of chalcone (0.004 mol), and thiourea (0.009 mol) in DMF (15 mL) was refluxed at 80 °C for 12 h in the presence of few drops of HCl. The progress of the reaction was monitored by TLC. After the completion of reaction, the reaction mixture was poured into ice water to give a precipitate that was filtered off and purified by column chromatography (40:50, diethyl ether-petroleum ether). The solid obtained was crystallized from ethanol. Yield: 77.6%; m.p. 166 °C; Anal. calc. for C_26_H_22_N_4_S_4_: C, 60.20, H, 4.27, N, 10.80, Found: C, 60.17, H, 4.23, N, 10.76; GC-MS *m/z* (rel. int.%): 520 (56) [M+1]^+^; IR (KBr) *v*_max_ cm^-1^: 2,960 (Ar-H), 1,651 (C=C), 1,590 (C=N), 1,134 (C-N), 678 (C-S); ^1^H-NMR (DMSO-*d*_6_) (δ/ppm): 7.75 (dd, 2H, Ar-H), 7.58 (dd, 2H, Ar-H), 7.51 (s, 2H, thiophene-H), 7.23 (s, 2H, Ar-Pym) 3.37 (s, 2H, SH), 2.58 (s, CH_3_), 2.36 (s, CH_3_); ^13^C-NMR (DMSO-*d*6) (δ/ppm): 184.93 (SH-C=N), 149.70, 145.61, 144.21, 142.80, 136.21, 134.74 (Ar-C), 14.57, 14.47 (CH_3_).

### 3.6. Organism culture and in vitro screening

Anti-bacterial activity was tested by the disk diffusion method with minor modifications. *S. aureus, S. pyogenes, S. typhimurium* and *E. coli* were subcultured in BHI medium and incubated for 18 h at 37 °C, and then the bacterial cells were suspended, according to the McFarland protocol, in saline solution to produce a suspension of about 10^-5^ CFU mL^-1^: 10 μL of this suspension was mixed with 10 mL of sterile antibiotic agar at 40 ºC and poured onto an agar plate in a laminar flow cabinet. Five paper disks (6.0 mm diameter) were fixed onto the nutrient agar plate. One mg of each test compound was dissolved in 100 μL DMSO to prepare stock solution from stock solution different concentration 10, 20, 25, 50, and 100 μg/μL of each test compound were prepared. These compounds of different concentration were poured over disk plate on to it. Chloramphenicol (30 μg/disk) was used as standard drug (positive control). A DMSO poured disk was used as negative control. The susceptibility of the bacteria to the test compounds was determined by the formation of an inhibitory zone after 18 h of incubation at 36 °C. **Table 1** reports the inhibition zones (mm) of each compound and the controls. The minimum inhibitory concentration (**MIC**) was evaluated by the macro dilution test using standard inoculums of 10^-5^ CFL mL^-1^. Serial dilutions of the test compounds, previously dissolved in dimethyl sulfoxide (DMSO) were prepared to final concentrations of 512, 256, 128, 64, 32, 16, 8, 4, 2 and 1 μg/mL. To each tube was added 100 μL of a 24 h old inoculum. The MIC, defined as the lowest concentration of the test compound which inhibits the visible growth after 18 h, was determined visually after incubation for 18 h, at 37 °C, and the results are presented in **Table 2**. Tests using DMSO and chloramphenicol as negative and positive controls were also perfomed.

## 4. Conclusions

A chalcone was prepared by the reaction of terephthalaldehyde with 3-acetyl-2,5-dimethyl-thiophene. Treatment of this chalcone with thiosemicarbazide/phenyl hydrazine/guanidine hydrochloride/thiourea afforded the corresponding pyrazoline, pyrazole and pyrimidine in good yields. The anti-bacterial activity of these compounds was examined using bacterial cultures and the results showed that the pyrazoline and pyrimidine showed increased antibacterial activity. Among the five compounds the pyrazoline derivative showed better anti-bacterial activity than the control drug chloramphenicol.

## Figures and Tables

**Table 1:** Antibacterial activity of the chalcone and its cyclized products, positive control chloramphenicol and negative control (DMSO) measured by the Halo Zone Test (unit, mm).

Compounds	Corresponding effect on microorganisms			
	*S. aureus*	*S. pyogenes*	*S. typhimurium*	*E. coli*
**1.1**	12.5 ± 0.3	11.6 ± 0.4	11.8 ± 0.2	12.2 ± 0.5
**1.2**	18.8 ± 0.5	19.5 ± 0.4	19.4 ± 0.4	21.8 ± 0.4
**1.3**	13.6 ± 0.5	13.2 ± 0.2	14.2 ± 0.2	14.5 ± 0.5
**1.4**	15.5 ± 0.4	15.8± 0.5	15.2 ± 0.4	16.2 ± 0.5
**1.5**	16.2 ± 0.2	16.6 ± 0.5	15.8 ± 0.4	16.8 ± 0.4
**Chloramphenicol**	17.0 ± 0.5	18.2 ± 0.4	17.2 ± 0.8	20.0 ± 0.2
**DMSO**	-	-	-	-

**Table 2:** Minimum inhibition concentration (MIC) of the chalcone and its cyclized products, positive control: chloramphenicol.

Bacterial Strain	MIC (μg mL^-1^) Compounds					
	1.1	1.2	1.3	1.4	1.5	Positive control
*Staphylococcus aureus*	128	32	128	32	64	32
*Streptococcus pyogenes*	128	32	64	64	64	32
*Salmonella typhimurium*	128	16	128	63	32	32
*Escherichia coli*	512	32	64	128	64	32
